# Recent Advances in Stimuli-Responsive Membranes: From Supramolecular Design to Controlled Permeability

**DOI:** 10.3390/membranes16030089

**Published:** 2026-02-28

**Authors:** Samanta Moffa, Serena Pilato, Michele Ciulla, Pietro Di Profio, Antonella Fontana, Fabrizio Masciulli, Gabriella Siani

**Affiliations:** 1 Dipartimento di Farmacia, Università “G. d’Annunzio”, Via dei Vestini 31, 66100 Chieti, Italy; samanta.moffa@unich.it (S.M.); serena.pilato@unich.it (S.P.); michele.ciulla@unich.it (M.C.); pietro.diprofio@unich.it (P.D.P.); antonella.fontana@unich.it (A.F.); fabrizio.masciulli@unich.it (F.M.); 2 UdA-TechLab, Research Center, Università degli Studi Gabriele d’Annunzio Chieti-Pescara, Via dei Vestini 31, 66100 Chieti, Italy

**Keywords:** stimuli-responsive liposomes, lipid bilayer dynamics, membrane permeability modulation

## Abstract

Stimuli-responsive liposomal membranes have attracted growing interest as dynamic soft materials capable of regulating permeability, fusion, and cargo release in response to external or internal triggers. By incorporating functional molecular or nanostructured guests, such as photochromic compounds, plasmonic nanoparticles, or ionizable lipids, bilayers can be endowed with reversible and tunable properties. These modifications often rely on the precise control of lipid packing, phase behaviour, and the formation of transient membrane defects that facilitate molecular transport. This review aims to provide an overview of the molecular design strategies and underlying mechanisms used to engineer such responsive liposomal systems, with particular emphasis on light- and heat-triggered behaviours and on supramolecular approaches that modulate membrane structure and dynamics. Emerging trends, current limitations, and opportunities for future development in functional lipid-based materials and biointerfaces will also be discussed.

## 1. Introduction

Achieving targeted and on-demand drug delivery remains one of the central challenges in modern nanomedicine and biomaterials science. The ability to control when, where, and how a therapeutic agent is released can dramatically improve treatment efficacy, minimize side effects, and reduce systemic toxicity. Among the different nanocarrier platforms developed to address this goal, liposomes stand out for their biocompatibility, structural versatility, and close resemblance to biological membranes [1,2,3,4,5]. Indeed, liposomes have long been recognized as versatile model systems, whose dynamic and self-assembled bilayers mimic key structural and functional aspects of biological membranes while enabling the study of membrane organization, permeability, and interactions with small molecules or nanostructures [6,7,8,9]. Their bilayer organization provides both a protective environment for encapsulated molecules and an adaptable interface that can be chemically or physically modified to respond to external or internal stimuli [10,11,12,13].

In recent years, the development of stimuli-responsive liposomal membranes has opened new perspectives for controlled drug release and membrane engineering. By incorporating responsive molecular units or nanoparticles capable of responding to light, temperature, pH, or other environmental changes, liposomes can be transformed into dynamic systems with reversible and tunable properties. These modifications enable precise regulation of permeability, fusion, and structural transitions, thereby linking fundamental membrane biophysics with practical biomedical applications.

A variety of approaches have been developed to confer responsiveness to liposomal systems. External physical stimuli such as light and heat can modulate membrane properties through direct energy input [14,15], while internal chemical stimuli, such as variations in pH or redox potential, exploit the intrinsic gradients present in biological environments [16,17].

Despite the large number of reports describing stimuli-responsive liposomal systems, several challenges remain. Quantitative correlations between molecular design, membrane composition, and macroscopic behaviour are often lacking, making it difficult to predict how a given modification will affect permeability or stability. In addition, direct comparisons across studies are complicated by variations in lipid composition, guest loading, and experimental conditions. A systematic understanding of how different classes of stimuli influence membrane organization is therefore essential to move from phenomenological observations to predictive design.

The present review aims to provide an overview of the principal classes of stimuli and the molecular mechanisms by which they alter liposomal membrane behaviour. We will discuss the physical and chemical stimuli most frequently used to induce structural or functional changes, focusing on light, heat, and pH. Understanding these fundamental relationships is critical for the rational design of next-generation responsive liposomes for controlled release, sensing, and biomedical applications.

Liposomal membranes can be engineered to respond to a wide range of external and internal stimuli, each acting through specific physicochemical mechanisms yet ultimately converging on the same outcome: a controlled perturbation of bilayer structure that modulates permeability, fluidity, or curvature.

Light is one of the most versatile external triggers because it offers high spatiotemporal precision, tunable wavelength selection, and easy reversibility. When photochromic molecules such as azobenzenes and spiropyrans are incorporated into the lipid bilayer, irradiation promotes reversible structural rearrangements that alter molecular geometry, polarity, and dipole moment, disturbing local lipid packing and modulating bilayer thickness and membrane fluidity [18,19,20,21,22,23].

At the macroscopic level, such molecular events may cause increased permeability, transient pore formation, or changes in the thermotropic phase behaviour of the bilayer. By selecting appropriate wavelengths and intensities, photoresponsive liposomes can therefore be used for reversible cargo release, fusion control, or sensing [24,25,26]. Nevertheless, challenges persist, particularly regarding the limited penetration depth of light in biological tissues and the need to minimize phototoxic effects. The development of red- or near-infrared-active chromophores, two-photon excitation, or up-conversion nanoparticles represents promising strategies to overcome these limitations [27,28,29].

Hyperthermic temperature (typically >40 °C) is another key driver of liposomal response. In thermosensitive liposomes (TSLs), controlled heating induces a gel-to-liquid crystalline transition in the lipid bilayer, resulting in enhanced permeability [30,31]. This can be achieved through direct heating or by embedding plasmonic nanoparticles, typically gold nanospheres or nanorods, that convert light into localized heat at their surface plasmon resonance [32,33,34,35]. Such photothermal effects can drive lipid phase transitions and transient pore formation, allowing for non-invasive and localized cargo release. However, achieving precise spatial control remains challenging due to heat diffusion and variations in nanoparticle distribution.

Among internal triggers, pH is especially relevant because pH gradients naturally occur in many physiological contexts, such as tumours, inflamed tissues, and intracellular compartments (endosomes, lysosomes). pH-responsive liposomes typically incorporate ionizable lipids or amphiphilic molecules that undergo acid-base equilibria [36,37]. Under acidic conditions, changes in charge and hydrogen bonding alter lipid packing and curvature, often destabilizing the bilayer or promoting fusion [38]. For example, formulations based on dioleoylphosphatidylethanolamine (DOPE) mixed with amphiphilic molecules containing a protonatable group, such as cholesteryl hemisuccinate, remain stable at neutral pH but readily convert into non-lamellar phases upon acidification [39]. Such behaviour has been widely exploited to achieve selective release in acidic environments, thereby improving targeting efficiency and therapeutic efficacy.

Overall, whether driven by external physical energy (light, heat) or environmental conditions (pH), these approaches share a unifying goal: to achieve precise, reversible, and tunable control over liposomal membrane structure and function. Understanding the interplay between molecular design and membrane response is essential for rational engineering of adaptive liposomal systems.

## 2. Mechanistic Framework: From Energy Input to Membrane Response

### 2.1. From Molecular Events to Membrane-Scale Responses

Stimuli-responsive liposomes operate through a cascade of interconnected processes in which a local perturbation, such as photon absorption, heat generation, or protonation, initiates a reorganization at the level of individual molecules, which then propagates through the membrane as structural and dynamical changes. Understanding this multiscale coupling is essential for the rational design of systems with predictable behaviour.

Photoactive molecules undergo electronic excitation followed by isomerization, cleavage, or conformational rearrangement; photothermal conversion in metallic nanoparticles produces localized heat that transiently disrupts lipid order; protonation of ionizable lipids modifies headgroup charge, hydration, and hydrogen bonding.

Although distinct in nature, these molecular events share a unifying characteristic: they perturb the delicate balance of van der Waals, electrostatic, and entropic forces that govern bilayer cohesion.

The cumulative effect of these local reorganizations becomes observable macroscopically as changes in fluidity, curvature, and permeability. The bilayer may soften, deform, or undergo transient pore formation, leading to controlled cargo release or vesicle fusion.

These cooperative phenomena illustrate how energy or chemical information encoded at the molecular level can be translated into functional macroscopic responses. Quantitative understanding of these couplings, supported by experimental and computational approaches, provides the foundation for designing truly adaptive liposomal systems.

### 2.2. Membrane-Scale Manifestations

One of the most universal responses to stimulation is the modulation of membrane fluidity. Alterations in the lateral packing of lipids affect the rotational and translational mobility of both lipid chains and embedded molecules. Photoisomerization or localized heating typically leads to a reduction in hydrocarbon chain order, accompanied by a decrease in bilayer thickness and an increase in the area per lipid [19,20,40].

Such fluidification enhances diffusion of small solutes and ions, facilitating permeability, although excessive disorder can compromise structural integrity. Achieving a balance between responsiveness and structural robustness remains a key challenge for practical applications.

Stimulus-induced variations in lipid packing can generate or relax membrane curvature. For example, the introduction of cis-azobenzene groups can promote the formation of invaginations, tubules, or budding structures [21,22,41].

Similarly, heating around plasmonic nanoparticles can create local curvature gradients due to thermal expansion, relevant for vesicle fusion [42].

Controlled release often arises from the creation of nanometer-sized transient pores or defects within the bilayer. Some studies have suggested that light or heat can promote the formation of defects that act as temporary diffusion pathways [43,44].

At higher levels of excitation or prolonged stimulation, accumulated stresses can exceed the bilayer’s elastic limit, driving irreversible transformations such as vesicle fusion, budding, or rupture [21,42]. While often considered detrimental for stability, controlled fusion or fission can be harnessed for targeted drug release, membrane trafficking studies, or artificial-cell construction.

Ultimately, the ability to predict and direct these large-scale responses represents a major frontier in the rational design of dynamic liposomal systems.

### 2.3. Experimental and Computational Approaches to Probing Stimuli-Responsive Membranes

A comprehensive understanding of how liposomal membranes react to external or internal stimuli requires the combination of complementary experimental and computational techniques, capable of resolving structural, dynamical, and thermodynamic changes across different time and length scales. Each method provides a distinct window on the complex coupling between molecular events and collective membrane behaviour.

Fluorescence spectroscopy remains one of the most widely used tools for monitoring changes in membrane organization and permeability. Fluorescent probes such as 1,6-diphenyl-1,3,5-hexatriene (DPH), Laurdan or pyrene report on lipid order and polarity through variations in emission spectra [8,9,45,46,47]. Förster resonance energy transfer (FRET) assays provide nanometer-scale sensitivity to lipid mixing and vesicle fusion [48], while encapsulated dyes like calcein, 5(6)-carboxyfluorescein, or sulforhodamine B provide complementary quantitative information on leakage and release kinetics [49,50,51].

Thermodynamic techniques such as differential scanning calorimetry (DSC) and isothermal titration calorimetry (ITC) offer direct access to the thermodynamics of phase transitions and interaction enthalpies [9,52,53,54]. Shifts in the main transition temperature (T_m_) or changes in transition cooperativity can reveal how photoisomerization, protonation, or nanoparticle heating perturb the lipid matrix.

Direct visualization methods contribute crucial structural information. Cryogenic transmission electron microscopy (cryo-TEM) and atomic force microscopy (AFM) capture stimulus-induced morphological transformations such as budding, pore formation, or vesicle fusion at nanometer resolution [55,56,57]. In situ irradiation or temperature control during imaging has made it possible to capture transient intermediate states of photo- or thermo-responsive liposomes [58].

Complementary scattering techniques, particularly small-angle X-ray scattering (SAXS) and small-angle neutron scattering (SANS), provide structural information on bilayer thickness, lamellarity, and phase coexistence [59]. Time-resolved SAXS/SANS experiments could track the kinetics of structural transitions upon light or heat stimulation, offering valuable quantitative parameters such as correlation lengths and relaxation times.

Computational modeling plays a crucial role in linking molecular design to macroscopic outcomes. All-atom and coarse-grained molecular-dynamics simulations bridge molecular design and mesoscale outcomes, reproducing structural rearrangements following photoisomerization, protonation, or local heating [6,60,61,62,63].

Taken together, these approaches form an integrated toolbox that connects molecular-scale perturbations to mesoscale membrane responses, providing the basis for mechanistic understanding and rational design of stimuli-responsive liposomal systems.

**Integrative and emerging approaches.** A more reliable picture of membrane responsiveness might arise from correlative studies that integrate multiple techniques. For instance, combining fluorescence and SAXS experiments under controlled illumination could help relate molecular disorder to potential structural rearrangements, while coupling calorimetry with MD simulations could provide a way to partially disentangle the enthalpic and entropic contributions to phase transitions.

Emerging techniques such as super-resolution microscopy and time-resolved X-ray photon correlation spectroscopy (XPCS) represent promising tools for future investigations. Although their application to liposomal systems is still limited, these methods could enable real-time observation of nanoscale remodeling and dynamic structural fluctuations, complementing established spectroscopic and scattering approaches [64,65].

Despite these advances, correlating molecular-scale observations with macroscopic functionality remains non-trivial. Differences in sample preparation, lipid composition, and stimulus delivery often complicate direct comparison between studies. Standardized protocols, real-time multi-modal measurements, and transparent data reporting will be essential to achieve reproducible and quantitative benchmarks. As experimental and computational techniques continue to converge, the field is moving toward predictive models capable of guiding the design of next-generation stimuli-responsive liposomes with tailored dynamic behaviour.

## 3. Thermo- and Photo-Responsive Liposomes Incorporating Gold Nanoparticles

Gold nanoparticles (AuNPs) embedded in or associated with liposomal membranes act as highly efficient local transducers of optical energy into heat. The physical processes that convert photon absorption into membrane perturbation operate across multiple time and length scales and determine whether cargo release proceeds mainly via thermal softening and phase transition, transient pore formation, or mechanical disruption. Understanding these mechanisms and how they depend on nanoparticle and membrane design is essential to rationally tune release kinetics, spatial selectivity, and biocompatibility.

**From light absorption to local heating.** AuNPs exhibit localized surface plasmon resonances (LSPR) whose spectral position, absorption cross-section, and nonradiative decay pathways depend strongly on particle size, shape, and dielectric environment [66]. Under illumination, typically in the NIR window for biological applications, the excited conduction electrons in gold nanoparticles undergo rapid electron-phonon coupling within a few femtoseconds to picoseconds, followed by phonon-lattice equilibration on picosecond-nanosecond timescales. This cascade of energy transfer results in a localized temperature rise at the nanoparticle surface, which subsequently dissipates into the surrounding solvent and lipid matrix [67,68]. The spatial extent of the thermal gradient generated by plasmonic nanoparticles depends strongly on particle size and the thermal conductivity of the surrounding medium. For isolated gold nanospheres, the heating remains essentially local, typically confined within a few tens of nanometres from the surface, whereas clustered or densely coated systems exhibit broader, overlapping thermal fields due to collective heat dissipation and plasmonic coupling [68,69,70].

Experimental studies by Xia et al. demonstrated that AuNP-liposome hybrid systems can achieve finely tunable photothermal temperature control, with temperature-sensitive responses occurring within fluctuations of less than 1 °C, enabling precisely regulated photothermal therapy [71]. Complementary findings by García et al. showed that this sensitivity is strongly dependent on membrane composition, as increasing cholesterol content enhances structural stability but raises the temperature threshold required for efficient release, highlighting the delicate balance between thermal responsiveness and bilayer rigidity [72].

**Two regimes of activation: continuous vs. pulsed illumination.** A useful practical distinction emerges between continuous-wave (CW) or long-pulse and short-pulse (ns-ps) laser regimes, as they engage distinct release mechanisms. Under CW or long-pulse illumination, heating occurs gradually, producing a quasi-equilibrium temperature rise within the bilayer. When the local temperature approaches or exceeds the phase-transition point (T_m_) of thermosensitive lipids or of embedded polymers, the membrane undergoes gel-to-liquid transitions accompanied by increased lateral diffusivity. These changes reduce packing order and transiently open defects that enhance permeability. Controlled, often reversible release has been consistently reported under CW/NIR illumination in DSPC (1,2-distearoyl-*sn*-glycero-3-phosphocholine) liposome-AuNP hybrid systems, where photothermal conversion drives mild, tunable heating of the bilayer [71]. Similarly, polymer-integrated Poly(N-isopropylacrylamide)-Au (PNIPAM-AuNP) liposomes exhibit synergistic photothermal and thermoresponsive behaviour, achieving adjustable release profiles without significant structural degradation [73]. Although CW irradiation supports reproducible and localized activation, it remains limited by heat diffusion and the potential for collateral warming of surrounding tissues.

In contrast, short-pulse (nanosecond to picosecond) laser excitation can induce extreme, highly localized non-equilibrium phenomena, including the formation of vapor nanobubbles (plasmonic cavitation) around AuNPs. The rapid expansion and collapse of these nanobubbles generate mechanical forces and transient pores that allow ultrafast release of encapsulated cargo, often within micro- to milliseconds, while keeping the bulk temperature rise modest. This mechanism is particularly effective for rapid endosomal escape and burst release, as documented for liposome-tethered AuNPs under pulsed NIR irradiation [74]. Because the thermal field dissipates within a few nanoseconds, structural integrity can often recover, making pulsed irradiation a promising strategy for on-demand, high-speed delivery. However, the magnitude and reproducibility of such effects are highly sensitive to nanoparticle size, aggregation state, and spatial distribution within or around the membrane. Further quantitative studies combining ultrafast spectroscopy and molecular dynamics simulations will be essential to disentangle thermal and mechanical contributions to pulsed light-induced release.

**Interaction between NPs and membrane.** The functional performance of gold nanoparticle-liposome hybrids depends critically on how the plasmonic component interacts with the lipid matrix. Beyond the fundamental photothermal mechanism, three structural factors, i.e., nanoparticle geometry, surface chemistry, and the surrounding lipid environment, collectively determine the efficiency, selectivity, and reproducibility of the liposomal response.

Nanoparticle geometry dictates the position and intensity of the localized surface plasmon resonance and therefore the efficiency of optical-to-thermal energy conversion. Spherical AuNPs (10–40 nm) are the most frequently employed due to their straightforward synthesis and high colloidal stability. However, morphologies such as nanorods, nanocubes, and nanostars exhibit stronger absorption in the biological near-infrared (NIR) window (700–900 nm) and higher photothermal conversion efficiencies. Comparative studies of different AuNP shapes interacting with model membranes revealed that rod-shaped AuNPs (AuNRs) achieve greater cellular uptake and enhanced photothermal conversion compared to cube-shaped or spherical particles [75]. Although the anisotropic geometry favours uptake and potential in photothermal therapy, its influence on membrane perturbation in terms of fluidity and packing order is not explicitly quantified in this work, and the improved performance must still be balanced with concerns about colloidal stability and aggregation in lipid-rich environments.

Particle size critically modulates both the optical response of gold nanoparticles and their mode of interaction with lipid bilayers. Experimental and theoretical studies have shown that the thermodynamic balance between insertion and adsorption is strongly influenced by nanoparticle diameter and surface chemistry. Ultra-small hydrophobic clusters (typically ≤10 nm) can embed within the bilayer core with minimal elastic penalty, directly perturbing acyl-chain order and local packing. In contrast, larger particles (>20–30 nm) preferentially adsorb or tether to the membrane surface, where they interact mainly through electrostatic or van der Waals forces and may promote local curvature or domain reorganization rather than full insertion.

This size-dependent behaviour has been rationalized through continuum and atomistic simulations, which show that the free energy of insertion increases steeply with particle diameter as lipid deformation and hydration penalties grow [76,77]. Systematic experiments corroborate this trend: Contini et al. identified two critical size thresholds for AuNP-membrane interaction, distinguishing small particles that partially penetrate from larger ones that remain surface-bound [78].

Complementary observations from Santhosh et al. demonstrated that hydrophobic AuNPs embedded in 1-stearoyl-2-oleoyl-*sn*-glycerol-3-phosphocholine (SOPC) bilayers locally disorder acyl chains, confirming that AuNPs can strongly interact with the lipid core while preserving membrane structural integrity, whereas excessive nanoparticle incorporation has been reported to disrupt the membrane and impair vesicle formation [45].

These findings highlight that embedded configurations favour efficient, highly localized heat transfer (beneficial for photothermal activation) but may compromise mechanical integrity, whereas larger tethered particles offer more predictable and reversible photothermal control at the cost of weaker coupling to the lipid interior.

Surface chemistry governs both colloidal stability and membrane affinity. Additives such as DDAB (didodecyldimethylammonium bromide) or DOTAP (1,2-dioleoyl-3-trimethylammonium-propane) can further modulate AuNP distribution by attracting or repelling charged surfaces. In particular, cationic liposomes exhibit strong electrostatic attraction toward AuNPs, facilitating adsorption and potential embedding, whereas anionic membranes interact less strongly, resulting in limited nanoparticle association at the bilayer surface [72,79].

The aggregation state of AuNPs itself affects plasmonic behaviour: interparticle coupling enhances absorption but also introduces heterogeneity and uncontrolled heating. Consequently, maintaining colloidal stability during synthesis and within lipid assemblies is crucial for reproducible thermal responses.

The composition and mechanical properties of the liposomal membrane play an equally decisive role in dictating the photothermal response. Factors such as cholesterol content, acyl-chain saturation, and the presence of charged or PEGylated lipids influence both membrane fluidity and thermomechanical response. Cholesterol, widely used to regulate bilayer order, increases mechanical rigidity and reduces passive leakage but raises the temperature required for photothermal release. In the study by García et al., increasing the cholesterol proportion in thermosensitive liposomal formulations influenced the doxorubicin release profiles observed at 37 °C and 42 °C, indicating that cholesterol content affects the balance between membrane stability and thermally triggered drug release [72].

Cryo-electron microscopy has provided direct structural insight into how the membrane phase state modulates AuNP-liposome hybrid formation. Comparative imaging of vesicles composed of fluid DOPC (1,2-dioleoyl-*sn*-glycero-3-phosphocholine) and gel-phase DPPC (1,2-dipalmitoyl-*sn*-glycero-3-phosphocholine) revealed markedly different nanoparticle organization at the membrane interface [80]. In DOPC systems, AuNPs adhered to the outer lipid shell and tended to form localized clusters without causing vesicle aggregation or disruption, consistent with plasmon coupling signatures observed spectroscopically. In contrast, AuNPs interacting with DPPC vesicles remained largely as individual particles but acted as intervesicular linkers, bridging adjacent liposomes into extended aggregates. These observations indicate that the degree of acyl-chain saturation, and thus membrane stiffness, governs not only nanoparticle clustering on the bilayer but also the supramolecular architecture of the resulting hybrids. Softer, liquid-crystalline membranes favour surface adsorption and local plasmonic coupling, whereas more rigid gel-phase bilayers promote crosslinking and network formation through AuNP bridging.

**Figure 1:**
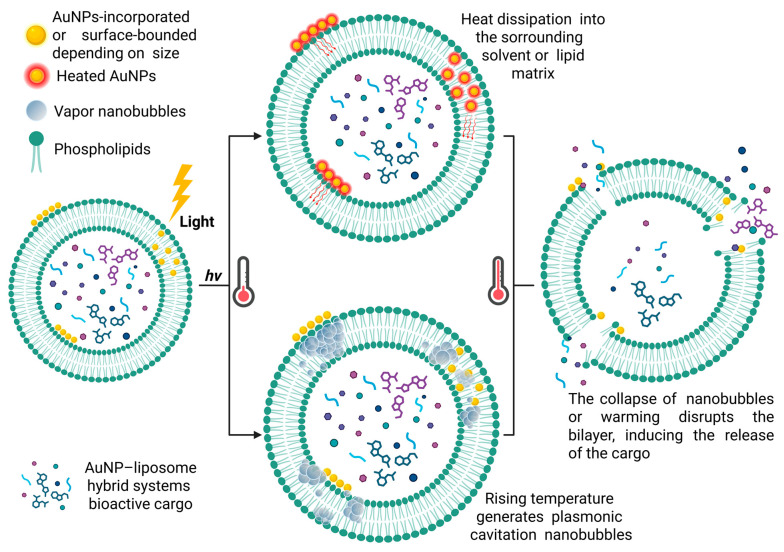
Schematic representation of AuNP-liposome hybrid systems and light-triggered release. Gold nanoparticles (AuNPs) embedded within or adsorbed onto the liposomal membrane absorb light and convert it into heat. Localized heating leads to membrane perturbation, which destabilizes the membrane, ultimately promoting the release of the encapsulated cargo.

In summary, Figure 1 provides an overview of the functioning of AuNP-liposome hybrid systems, highlighting the role of plasmonic heating in the light-triggered release of the encapsulated cargo.

**Functional outcomes and useful metrics.** Photothermal activation may lead to several functional outcomes, such as reversible permeability increase desirable for repeated on-off release cycles; transient pore formation/burst release, useful for rapid cargo delivery or endosomal escape; irreversible rupture, sometimes acceptable for single-use burst delivery but of limited reusability.

To enable consistent evaluation across different photothermal systems, certain standardized metrics are particularly informative and should be reported and compared across studies: incident wavelength and power density (mW·cm^−2^), pulse duration (if pulsed), local ΔT at the membrane (where measured), threshold fluence for onset of release, percent release (ON vs. OFF), response time, and number of reversible cycles without significant fatigue.

From a translational perspective, two considerations dominate: minimizing collateral thermal damage and addressing persistence/clearance of gold nanomaterials. CW protocols that require high fluences raise concerns for surrounding tissues; pulsed protocols avoid bulk heating but may cause mechanical damage. Furthermore, AuNP biodistribution and long-term fate (clearance vs. accumulation) depend on size, shape and surface chemistry, and must be balanced against the therapeutic gains of precise photothermal control.

**Critical considerations.** A concise comparison of the most relevant experimental systems reported is provided in Table 1. The works were selected to represent the main design paradigms currently explored for gold nanoparticle-liposome hybrids, covering differences in nanoparticle configuration (templated, tethered, or embedded), lipid composition, external stimulus, and release mechanism.

The literature reveals that the apparent diversity of AuNP-liposome systems converges on a few unifying design principles. Stronger nanoparticle-membrane coupling enhances photothermal responsiveness but increases the risk of destabilization; greater membrane rigidity or higher cholesterol content improves colloidal stability while diminishing heat sensitivity; and geometric anisotropy amplifies light absorption and conversion efficiency at the expense of reproducibility.

Beyond drug delivery, AuNP-liposome hybrids offer exciting opportunities in biosensing, mechanobiology, and artificial-cell design, where their ability to transduce optical energy into mechanical or chemical outputs could be harnessed to probe membrane elasticity, trigger fusion events, or construct optically addressable protocells. Overall, these systems represent the convergence of plasmonic nanoscience and membrane biophysics: from early proof-of-concept models, they have evolved into tunable nanoscale tools for precise energy conversion and controlled release. Yet their translation from conceptual prototypes to reliable biomedical platforms will depend on advancing quantitative thermal mapping, rational interface engineering, and rigorous biocompatibility assessment-key steps toward establishing predictable, multifunctional photothermal liposomes.

## 4. Photoresponsive Liposomes Based on Molecular Photoswitches

Light offers a unique means of remotely controlling the structure and permeability of liposomal membranes with high spatial and temporal precision [18,81,82]. The integration of photochromic molecules enables the design of light-responsive membranes whose organization and permeability can be reversibly modulated by irradiation. Unlike photothermal systems, photochromic compounds transduce photon energy directly into conformational and electronic changes, producing controlled variations in membrane fluidity, curvature, and phase behaviour. A key advantage of photoresponsive liposomes lies in the non-contact, reversible, and programmable nature of light activation, which can be patterned in time and space to achieve precise control over membrane function.

Recent progress in molecular design, particularly in visible- and near-infrared-active switches, has greatly improved compatibility with biological environments [83,84,85,86,87]. However, efficient operation within the confined, heterogeneous membrane matrix remains challenging, as steric constraints and restricted mobility can reduce photochemical quantum yields. Among the different classes of investigated photoswitches, azobenzenes and spiropyrans have emerged as the most robust and versatile. Their well-understood, reversible transformations make them ideal molecular actuators for studying how light regulates permeability and transport in self-assembled membranes. Together, these systems exemplify how molecular photoswitches can impart direct photomechanical functionality to lipid membranes, providing a non-invasive and precisely tunable platform for controlled release and photochemical modulation.

### 4.1. Azobenzene-Based Liposomes

Among organic photoswitches, azobenzenes remain the most extensively investigated for imparting light-responsiveness to liposomal systems [24,88]. Their reversible trans-to-cis photoisomerization induces substantial changes in molecular geometry, dipole moment, and polarity, which in turn perturb the local lipid packing and alter bilayer fluidity, curvature, and permeability. Depending on the mode of incorporation, i.e., covalently attached to phospholipids, intercalated as amphiphilic guests, or assembled into supramolecular nanoclusters, azobenzene derivatives can act as molecular actuators that couple photon energy to mechanical motion at the membrane level.

The response of azobenzene-containing liposomes arises from the delicate interplay between the photochemical properties of the switch and the behaviour of the bilayer. In early studies, simple aryl-azo derivatives embedded as small guests within phosphatidylcholine bilayers demonstrated that reversible photoisomerization could modulate the permeability to encapsulated fluorophores and small ions. The work by Siani et al. exemplifies this principle, showing that aryl-azo derivatives of thymol, when inserted into POPC (1-palmitoyl-2-oleoyl-*sn*-glycero-3-phosphocholine) liposomes, induce reversible changes in membrane permeability upon alternating irradiation at 365 and 430 nm [89]. The degree of switching was finely tunable by illumination wavelength and power, correlating with chloride transport and fluorescence-detected release kinetics. These systems highlight the feasibility of modulating passive transport by nanoscale reorganization of the bilayer without the need for covalent lipid modification.

Incorporation through covalently bound photolipids represents a more structurally defined approach. Photolipids such as azo-phosphatidylcholines (azo-PCs) integrate the azobenzene unit directly within the hydrophobic tail, enabling efficient transmission of conformational change to the surrounding lipids. The resulting vesicles (azosomes) display ultrafast, reversible cargo release under alternating illumination at 365 and 455 nm, within seconds (<3 s), and repeatable switching over multiple cycles [90]. Functional validation in neuronal cultures demonstrated the capacity for optically controlled neurotransmitter release and neuromodulation, establishing azosomes as powerful model systems for precision photochemical control in biological environments.

A third, rapidly growing strategy involves the supramolecular assembly of azobenzene units at the membrane interface. Nanocluster-based architectures, such as azobenzene-modified nanoclusters tethered to liposomes, amplify the collective photomechanical response by promoting cooperative reorganization of the lipid matrix [91]. In these *LipCal@NC* hybrid systems, light-induced isomerization not only alters the molecular dipole but also modulates structural modification of the membrane, in agreement with molecular dynamics simulations showing enhanced lipid disorder and lateral diffusion in the cis state, increasing the cargo release from ~33% to ~47% after 360 min of UV irradiation.

Indeed, azobenzene-induced mechanical remodeling of vesicles has been directly visualized using optical microscopy, showing that light-induced isomerization of azobenzene amphiphiles can trigger large-scale shape transformations, including budding, elongation, and reversible curvature inversion [21]. These findings link the molecular-scale event of dipole rotation to mesoscale changes in vesicle morphology, providing an experimental framework to link photoisomerization dynamics with mechanical response.

Mechanistic studies further rationalize these improvements by revealing how lipid environments (packing and polarity) modulate the balance between isomerization pathways, ultimately influencing switching efficiency and stability [92].

Chemical modification of the azobenzene core offers a further degree of control over photo-response. Substituent effects on electronic distribution and torsional freedom can adjust both the rate of trans-to-cis conversion and the stability of the cis isomer within the membrane [89,93]. Zhang et al. systematically varied the electronic nature of substituents and demonstrated that substituents with a positive charge and strong electrophilicity significantly reduced the photoisomerization ratio in liposomal systems [94]. Depending on substituent and irradiation protocol, release ranged from 12% to 18% over 12 h, showing how electronic modulation can fine-tune the balance between responsiveness and structural stability.

The introduction of mechanically active azobenzene lipids capable of rotational or bending motion under light excitation has led to vesicles that exhibit “nanomechanical” action, aiding endosomal escape and cytosolic delivery. Recent work has demonstrated that incorporating cationic azobenzene lipids into DSPC-based liposomal membranes enables light-controlled drug release and efficient intracellular delivery [88]. Upon irradiation, the reversible trans-to-cis isomerization of the azobenzene units induces continuous molecular reorientation within the bilayer, transiently increasing membrane permeability and promoting doxorubicin release. Once internalized, these photoresponsive liposomes exert a nanomechanical destabilization effect on endosomal membranes, facilitating cytosolic escape and enhancing antitumour efficacy. In vivo, co-encapsulation of up-conversion nanoparticles (UCNPs) to convert NIR into UV-visible emission further improved activation efficiency, leading to significant tumour growth inhibition under NIR illumination [88].

Building upon this concept, other studies have also explored the integration of azobenzene derivatives with up-conversion nanoparticles to overcome the limited tissue penetration of UV light. For instance, a core–shell UCNP system incorporating an azobenzene-containing molecule (Azo-PSG) enabled light-triggered disassembly of doped liposomes under NIR excitation. The up-conversion architecture allows efficient cis-to-trans photoisomerization and controlled drug release upon NIR irradiation. This strategy effectively extends the operational window of photoresponsive liposomes into biologically compatible wavelengths, broadening their applicability in light-controlled nanocarriers [95].

Beyond their role as membrane-disrupting photoswitches, azobenzene derivatives have also been incorporated into synthetic ion channels, offering a more biomimetic route to light-gated transport. Several recent studies illustrate how supramolecular design can achieve photo-controllable ionic conduction across lipid bilayers without compromising structural integrity [89,96,97,98].

For instance, the artificial K^+^-selective channel P3 integrates azobenzene photoisomerization with β-cyclodextrin host-guest chemistry to generate multiple transport states (“ON”, “partially OFF”, “totally OFF”) in response to alternating 365 and 450 nm light [96]. This light-driven modulation of supramolecular interactions enables precise control over ion flux in both model membranes and living cells, leading to K^+^ depletion, mitochondrial depolarization, and apoptosis, a mechanism with potential anticancer implications. Similarly, azo-functionalized bambusuril derivatives (azo-BUs) demonstrate reversible photo-control of Cl^−^ transport activity through E-Z isomerization. These systems exemplify the dual regulatory capacity of functioning as light-tunable therapeutic agents [97].

Complementary studies on azobenzene-fused bis(1,3-diol) molecules further confirm that photoisomerization can reversibly gate Cl^−^/NO_3_^−^ antiport through self-assembled channels within lipid bilayers. Conductance and theoretical analyses support the formation of discrete, supramolecular channels capable of dynamic switching between open and closed states under 365/450 nm irradiation [98].

Together, these studies mark an important evolution from simple permeability modulation toward programmable, biomimetic ion transport, demonstrating how azobenzene photoisomerization can bridge the gap between synthetic membrane engineering and light-regulated ion transport resembling that of biological channels.

A comprehensive overview of representative systems and their functional outcomes is provided in Table 2.

Although azobenzene-based photoresponsive liposomes have achieved notable progress, the current literature still reveals substantial variability and methodological fragmentation that complicate cross-study comparisons and the derivation of general design principles. A critical examination of the studies summarized in Table 2 highlights several recurrent issues.

Reported release percentages span from rapid (<3 s) to slow (hours) regimes depending on assay format and irradiation conditions. Variability in chromophore loading, light fluence, and lipid formulation prevents straightforward comparison. Normalization of data to absorbed photon dose or chromophore concentration is rarely applied but essential for cross-laboratory benchmarking.

Several mechanistic studies show that the viscosity and restricted mobility of lipids significantly reduce isomerization quantum yields. These effects reduce the extent of molecular switching but enhance mechanical responses in the membrane, indicating that molecular design should balance chromophore mobility with sufficient anchoring to the bilayer.

Leakage or release can originate from local disordering, transient pore formation, or small-scale phase transitions. Differentiating photoisomerization-driven events from thermal contributions, especially in UCNP-assisted systems, remains a persistent problem. Integrating optical activation with calorimetry, fluorescence lifetime imaging, and MD simulations would clarify causality.

Most azobenzene systems still require UV or blue light, which limits penetration and raises phototoxicity. Red-shifted designs and two-photon or UCNP-based strategies extend operability but introduce new issues of complexity and potential cytotoxicity.

Establishing quantitative descriptors, such as permeability coefficients normalized to chromophore content, or bilayer relaxation times, would permit predictive modelling and yield transferable design rules.

In summary, azobenzene-based photoresponsive liposomes have transitioned from conceptual prototypes to versatile, multifunctional systems, yet predictive control remains elusive. Standardized quantification, mechanistic rigor, and red-shifted biocompatible designs are essential to transform these model systems into platforms for controlled release, sensing, and biomedical photomodulation.

### 4.2. Spiropyrans-Based Photoresponsive Systems

Spiropyrans (SPs) represent a second major class of photochromic molecules employed to impart light sensitivity to lipid membranes. They undergo a reversible isomerization between a closed, nonpolar spiropyran (SP) form and an open, zwitterionic merocyanine (MC) form upon optical or thermal stimuli. This transformation involves cleavage of the C-O spiro bond and a reorganization of charge distribution and dipole moment, leading to pronounced changes in molecular polarity and hydrogen-bonding capacity. When embedded within liposomal bilayers, these transitions translate into light-controlled variations in bilayer hydration, packing density, and permeability.

The duality between the hydrophobic SP form and the amphiphilic MC form makes spiropyrans particularly versatile for membrane-based applications. In the SP configuration, spiropyrans reside mainly within the hydrophobic core of the bilayer, contributing minimally to disruption. Upon UV or blue-light irradiation, conversion to the polar MC form increases local hydration and induces stress at the lipid-water interface, leading to transient perturbations that facilitate the diffusion of encapsulated solutes or ions across the membrane. The process is reversible: visible-light irradiation or thermal relaxation restores the SP form and re-establishes the membrane barrier.

A representative overview of the most relevant spiropyran-based photoresponsive liposomal systems is provided in Table 3.

A particularly illustrative example of spiropyran-based photoactuation is provided by Zappacosta et al., who showed that embedding SP in POPC bilayers enables light-controlled proton transport [99]. Upon UV irradiation and in the presence of protons, SP converts to its protonated merocyanine form (MEH^+^), which migrates toward the membrane-water interface. Subsequent visible-light irradiation regenerates SP and releases the captured protons into the vesicle lumen. Although the overall flux is low (≈1–2%), the system operates as a minimal photoactivated ion-gating device capable of lowering intraliposomal pH through reversible SP ⇌ MEH^+^ cycling. This study highlights the ability of spiropyrans to couple photoisomerization with directional ion movement, expanding their role beyond simple permeability modifiers and underscoring their potential for designing light-gated delivery or protocellular systems.

Recent advances demonstrate that spiropyrans can be engineered to respond not only to UV light but also to NIR excitation, greatly expanding their biomedical applicability. Across different molecular designs, these systems exploit the reversible SP → MC photoisomerization to regulate hydrophobicity, fluorescence, morphology, and drug release, often in combination with pH sensitivity.

A first strategy involved molecular fusion of spiropyrans with other chromophores, as exemplified by the spiropyran-coumarin-chlorambucil (SP-Cou-Cbl) conjugate [100]. Here, the acidic tumour environment promotes spyropyran ring-opening and fluorescence activation of the coumarin unit, enabling tumour localization, while externally applied light triggers-controlled drug release. This “dual-key” mechanism, pH unlocking plus light-regulated release, offers both diagnostic readout and spatiotemporal control. Importantly, the system retains good biocompatibility and enables dose-dependent photoactivation in cancer cells.

A second design motif exploits supramolecular metal-spiropyran assemblies. In the merocyanine-Zn-acetylsalicylic acid (ASA) ternary complex, visible light modulates the coordination geometry and thus the release of the pharmaceutically active compound [101]. This approach illustrates that spiropyrans can function not only as switches but also as structural elements in multi-component drug assemblies, enabling light-controlled, multi-payload delivery. The visible-light responsiveness and reversibility further highlight the feasibility of spiropyran-based systems for externally triggered activation using clinically safer wavelengths.

Most advanced strategies integrate spiropyran-functionalized amphiphilic polymers with up-conversion nanoparticles (UCNPs) to enable true NIR responsiveness [102]. In these nanocomposites, NIR irradiation is converted into UV/visible light inside the particle core, inducing SP → MC conversion, polymer swelling, and morphological disruption of the nanostructure. This switching is synergistic with acidic pH, which also protonates SP to MC, producing strong dual-stimulus responsiveness. Release of hydrophobic dyes and doxorubicin is markedly enhanced upon NIR irradiation, and the system may achieve efficient tumour-cell killing with minimal phototoxicity. This demonstrates the potential of UCNP-assisted spiropyran activation for deep-tissue applications, overcoming the inherent tissue penetration limits of UV-visible light.

Together, these systems highlight a broader conceptual trend: exploiting both environmental (pH) and remote (NIR) triggers to achieve selective drug release with high spatiotemporal precision. Despite their distinct architectures, all three studies underscore the versatility of the SP/MC equilibrium and point toward hybrid designs where pH-photo cooperativity, multi-payload delivery, and deep-tissue optical control converge in next-generation smart nanocarriers.

Recent work has extended spiropyran photoresponsiveness to membrane-active peptides by incorporating a spiropyran-derived amino acid into the BP100 peptide [103]. Light-driven SP ⇌ MC switching was shown to modulate peptide conformation and, consequently, its ability to perturb membranes. The hydrophobic SP state-maintained helicity and membrane activity, whereas the polar MC form weakened amphiphilicity and reduced cytotoxicity. These results demonstrate that spiropyrans can provide reversible optical control over peptide–membrane interactions, offering a promising strategy for developing light-regulated bioactive peptides and future photo-pharmacological tools.

Hybrid spiropyran-lipid and spiropyran-polymer systems further broadened the scope of photoactuation.

A recent spiropyran-based block copolymer system demonstrates how photochromic units can be integrated into multifunctional nanocarriers for combined diagnosis and therapy. The amphiphilic polymer undergoes reversible SP ⇌ MC switching under UV/visible light, enabling optical control over assembly, polarity, and drug release. In triple-negative breast cancer models, the MC state provided sensitive Cu^2+^ detection, while the polymeric nanostructures supported pH-enhanced and light-modulated DOX release together with efficient gene delivery [104]. The platform showed high transfection efficiency, strong anticancer activity, and good hemocompatibility, illustrating how spiropyran-containing polymers can serve as dual diagnostic and therapeutic (theranostic) carriers with externally tunable behaviour.

A recent example of spiropyran-based molecular control comes from shell cross-linked micelle (SCM) nanoreactors engineered to respond to both light and temperature [105]. In these systems, a photoswitchable spiropyran cross-linker and a thermoresponsive poly(2-oxazoline) corona cooperate to modulate the hydrophobicity and accessibility of different layers of the micelle. UV irradiation induces SP → MC conversion within the cross-linked shell, favouring transport of less hydrophobic substrates, whereas heating collapses the outer corona, selectively gating the entry of more hydrophobic molecules. This dual gating enables tunable, stimulus-specific substrate selectivity in catalytic asymmetric transfer hydrogenation performed in water. Although developed for catalysis, these SCMs illustrate a general design principle highly relevant to stimuli-responsive delivery systems: orthogonal photo- and thermo-switches can create hierarchical transport channels, reminiscent of biological compartmentalization. Such architectures highlight how spiropyran units can be embedded not only to disrupt or permeabilize membranes, but also to encode controlled, layered accessibility in soft nanocarriers.

### 4.3. Comparative Analysis for Photoresponsive Liposomes

The body of work summarized above highlights two complementary paradigms for the optical control of lipid membranes. Azobenzenes operate predominantly as mechanical actuators: their trans-to-cis isomerization produces marked geometrical/structural changes that can translate into rapid modifications of packing density, curvature, and membrane tension. In contrast, spiropyrans tend to act primarily through interfacial and electrostatic modulation, where SP ⇌ MC switching alters polarity, hydration, and dipole moment, often generating smoother and more environmentally sensitive responses than azobenzenes. Importantly, these mechanisms should be viewed as dominant rather than exclusive, as both switches may exert mixed mechanical and interfacial effects depending on membrane composition and confinement.

**Figure 2:**
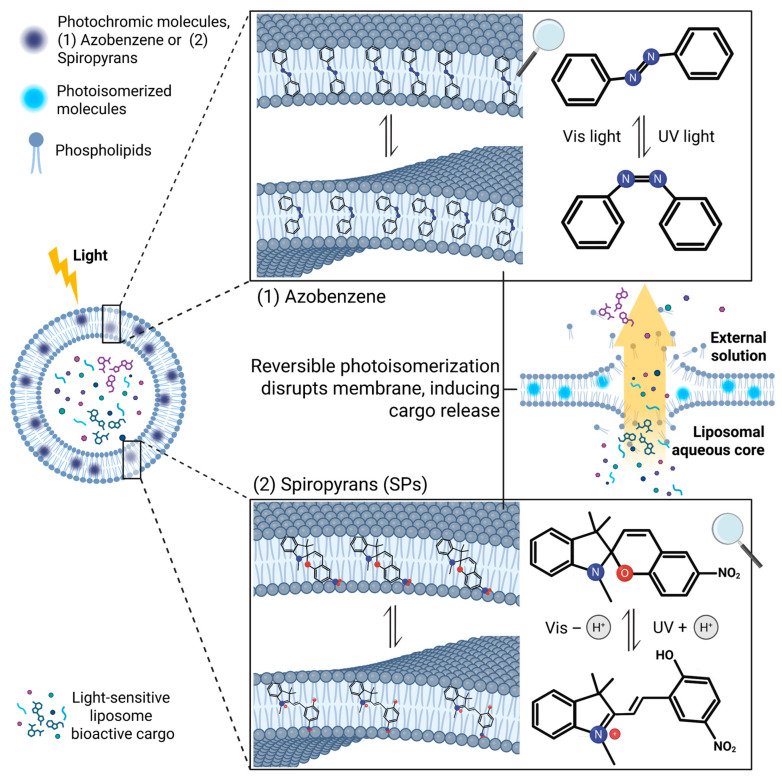
Light-responsive liposomes incorporating molecular photoswitches. Azobenzene-based switchable lipids undergo trans-to-cis isomerization that produces marked geometric/structural changes within the bilayer, acting predominantly through mechanical perturbation of packing, curvature, and membrane tension. Spiropyrans modulate permeability mainly through polarity- and charge-redistribution associated with SP ⇌ MC interconversion, resulting in smoother and more environmentally sensitive responses. These modes of actuation are dominant but not mutually exclusive: both switches can contribute simultaneous mechanical and interfacial effects and may ultimately be combined to achieve multi-level control over light-regulated cargo release.

This conceptual balance is illustrated in Figure 2, which compares the mechanically driven perturbation of azobenzenes with the polarity-dependent membrane disruption induced by spiropyrans, ultimately enabling light-controlled cargo release.

These contrasting behaviours translate into distinct operational advantages. Azobenzenes offer fast, high-amplitude responses, ideal for short-timescale deformation and burst-release. Spiropyrans provide tunability, pH/photo cooperativity, and compatibility with deeper-tissue activation (especially with UCNPs), making them well-suited to reversible, low-impact control.

Looking ahead, understanding how these modes of actuation unfold within lipid bilayers provides a framework for tailoring performance to application. Hybrid systems incorporating both photoswitches could enable multi-level regulation, where rapid azo-driven gating is complemented by slower, polarity-mediated modulation from spiropyrans, a synergistic approach reminiscent of the layered responsiveness of biological membranes.

Table 4 summarizes the key mechanistic and functional distinctions between the two photoswitch classes.

Despite the rapid evolution of azobenzene- and spiropyran-based strategies, several fundamental challenges remain. A major unresolved issue is the absence of quantitative, predictive relationships linking photochemical conversion efficiency to macroscopic membrane responses such as permeability, transport rates, or vesicle remodeling. In most systems, molecular photoisomerization is well characterized, while the downstream collective behaviour of the bilayer remains difficult to model, particularly when multiple photoactive dopants interact cooperatively within complex lipid compositions.

Addressing these gaps will require integrated methodologies that combine in situ optical spectroscopy, calorimetry, high-resolution imaging, and multiscale molecular simulations. Such approaches will be essential to determine how photoisomerization modulates bilayer mechanics, hydration, and tension, and how these changes propagate to functional outcomes such as controlled release or ion transport.

Ultimately, azobenzenes and spiropyrans should not be viewed as competing paradigms but as complementary extremes along a continuum of photochemical control. Azobenzenes provide rapid, geometry-driven mechanical actuation, while spiropyrans offer polarity-driven, environment-sensitive modulation. Together, they define a versatile design space for constructing programmable, light-gated nanocarriers and biomimetic soft materials capable of hierarchical and finely tunable responses. Their integration in hybrid architectures could represent the next step toward truly adaptive photoresponsive membranes for precision therapeutics.

Other photo-triggered strategies for modulating liposome stability, such as photodynamic lipid oxidation mediated by reactive oxygen species (ROS), have also been reported [106,107,108,109]. These strategies rely on ROS-mediated chemical modifications of lipids: hydroperoxide formation can transiently increase water permeability, while longer illumination can generate aldehyde products that destabilize the bilayer, potentially leading to vesicle collapse [106]. Compared with photothermal and photoswitch-based systems, ROS-mediated mechanisms are generally less predictable and lack the rapid, fully reversible switchability of the latter, and are therefore only briefly mentioned here. In general, ROS-responsive liposome stability must be carefully tuned to survive circulation while becoming activated only within a specific ROS concentration window, highlighting the challenges in achieving precise control under physiological conditions [107]. Recent work demonstrates that ROS-responsive liposomes can achieve targeted drug release in pathological microenvironments, showing that controlled activation is possible under specific conditions [108].

For example, cationic 1,2-dioleoyl-3-trimethylammonium-propane (DOTAP) incorporated into porphyrin–phospholipid (PoP) liposomes can accelerate near-infrared (NIR) light-triggered drug release via ROS-mediated lipid oxidation [109]. This effect enhances delivery to tumour endothelial cells and enables synergistic chemo-phototherapy effects. However, release depends on local ROS generation, light exposure, and interactions with biological barriers, further limiting precise and reversible control compared with photothermal and photoswitch-based systems.

## 5. pH-Responsive Liposomes

pH variations represent one of the most physiologically relevant internal stimuli exploited to control liposomal behaviour. Distinct extracellular and intracellular compartments exhibit characteristic pH values, from ~7.4 in the bloodstream to ~6.2–6.8 in tumour microenvironments, and down to ~5.0 in endosomes and lysosomes. Exploiting these natural pH gradients enables the development of liposomal systems that can undergo site-specific and self-activated release without the need for external stimuli, a feature of particular relevance for targeted drug delivery and precision oncology.

The mechanism underlying pH-responsiveness lies in the protonation-deprotonation equilibria of ionizable lipid headgroups or functional groups embedded within the bilayer. These reversible acid-base processes alter charge distribution, hydrogen bonding and hydration, thereby modulating bilayer packing, curvature stress, and mechanical stability. In acidic conditions, protonation typically reduces surface charge repulsion of prevailingly negatively charged liposomes, leading to tighter lipid packing and transitions from lamellar to nonlamellar phases. Such reorganizations can transiently increase permeability or trigger vesicle fusion and content release.

Classical examples include DOPE (1,2-dioleoyl-*sn*-glycero-3-phosphoethanolamine)-CHEMS (cholesteryl hemisuccinate) formulations, where protonation of CHEMS disrupts the lamellar bilayer stabilized at neutral pH, driving DOPE toward its inverted hexagonal phase and inducing rapid drug release [110]. Recent studies have extended this principle to a variety of weakly acidic amphiphiles with finely tunable pK_a_ values tailored for specific biological compartments [111,112,113].

Such design versatility enables the creation of nanocarriers capable of bypassing biological barriers, enhancing endosomal escape, and achieving stimuli-synchronized release profiles.

**Figure 3:**
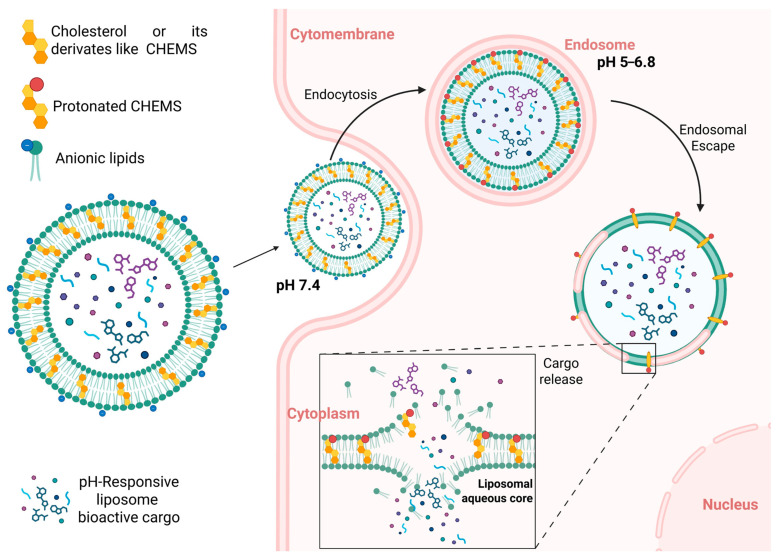
Intracellular mechanism of pH-responsive liposomes. Liposomes incorporating cholesterol derivatives (e.g., CHEMS) remain stable at physiological pH (≈7.4), but upon endocytosis, their environment becomes progressively acidic (pH 5–6.8). Protonation of CHEMS reduces lipid packing and increases membrane destabilization, promoting endosomal escape. Subsequent bilayer disruption in the cytoplasm enables diffusion of the encapsulated payload from the liposomal core into the surrounding medium.

Figure 3 illustrates how endosomal acidification protonates cholesterol derivatives such as CHEMS, weakens membrane stability, and ultimately triggers cytosolic cargo release.

Overall, pH-responsive liposomes exemplify how small molecular-scale changes in protonation and hydration can drive large-scale structural and functional transformations. Establishing quantitative links between lipid composition, pK_a_ tuning, and dynamic morphology remains a key challenge for predictive control. The following sections will examine representative formulations, mechanistic insights, and emerging trends in the application of pH-sensitive liposomes for targeted therapy and diagnostic use.

### 5.1. Molecular Mechanisms of Proton-Induced Membrane Remodeling

At the molecular level, the response of pH-sensitive liposomes is governed by how protonation events propagate through the membrane. Protonation of carboxylate or phosphate groups alters hydration layers and electrostatic forces. These changes perturb lateral pressure and membrane curvature. Molecular-dynamics simulations and experimental studies reveal that such perturbations can induce phase separation, pore formation, or membrane thinning, all of which facilitate solute transport [114,115,116]. Indeed, once a critical fraction of ionizable lipids becomes protonated, collective membrane reorganization takes place, leading to a sharp increase in permeability.

For example, studies on DOPE-CHEMS and related phosphatidylethanolamine systems show that upon acidification, protonation of the CHEMS headgroup decreases its effective charge and hydration, thereby reducing electrostatic repulsion at the lipid-water interface. As the cross-sectional area of the sterol-based hydrophobic region remains essentially unchanged, this imbalance shifts the effective molecular geometry from a bilayer-forming cylindrical shape toward an inverted cone. Lipids with this geometry preferentially adopt the H_II_ phase [117]. Similarly, the incorporation of weakly basic amphiphiles such as imidazole or histidine-modified lipids enables tunable protonation equilibria within the pH 5–6.5 range, ideal for targeting endosomal escape [111,112,118,119].

### 5.2. Functional Implementations and Biological Applications

Building on these mechanistic principles, the development of pH-sensitive liposomes has provided a novel approach for targeted cancer therapy. Such systems can achieve tumour-specific drug release, with minimal drug leakage at physiological pH and increased release under acidic tumour conditions, leading to improved cytotoxicity against cancer cells and enhanced cellular uptake. In addition, these carriers exhibit good biocompatibility and reduced systemic toxicity, making them promising platforms for chemotherapeutic delivery [120,121,122,123]. For instance, cisplatin-loaded DOPE-CHEMS liposomes have demonstrated an effective balance between stability in systemic circulation and responsiveness to acidic conditions. They remained stable at physiological pH (7.4) but released up to ~65% of cisplatin at pH 6.5 and over 80% at pH 5.5, resulting in enhanced cytotoxicity in cancer cell lines compared to free cisplatin, while maintaining good biocompatibility in acute toxicity studies [124]. Similarly, fucoidan-coated gemcitabine-loaded liposomes targeted pancreatic cancer cells, where fucoidan modification enhanced cellular uptake, increased cytotoxicity, and promoted drug accumulation at the tumour site. The pH-sensitivity of these liposomes facilitated endosomal escape, further enhancing tumour-specific drug release and minimizing systemic toxicity [125]. In parallel, glycopeptide-modified liposomes have exploited acid-cleavable linkers to expose antigens under acidic conditions, thereby promoting cross-presentation and cellular immune activation [126].

More recently, hybrid systems combining pH-sensitive liposomes with extracellular vesicles (EVs) have demonstrated further advantages. Pegylated pH-sensitive liposomes fused with EVs derived from MDA-MB-231 breast cancer cells and loaded with doxorubicin (HF-pHSL/EV-DOX) exhibited excellent chemical and physicochemical properties, including high encapsulation efficiency, small size, and good storage stability [127]. Importantly, the integration of EVs did not impair pH responsiveness, allowing effective tumour-specific drug release. In vitro studies showed cytotoxicity across breast cancer cell lines of multiple molecular subtypes and anti-migratory effects, while preliminary in vivo toxicity assessments indicated a favourable safety profile. Together, these findings position EV-liposome hybrids as a versatile and biologically compatible platform for multimodal cancer therapy.

Recent hybrid constructs, such as pH-responsive liposomes co-assembled with amphiphilic ruthenium-based oxygen probes, further demonstrate how therapeutic delivery can be combined with diagnostic imaging within a single platform; in these systems, pH sensitivity enhances tumour accumulation and cellular uptake, while oxygen-sensitive probes enable ratio-based fluorescence imaging [120].

Collectively, these studies demonstrate that pH-sensitive liposomal formulations can improve therapeutic outcomes, enhance tumour targeting, and reduce off-target effects, highlighting their potential as next-generation drug delivery platforms in oncology.

Representative formulations and experimental outcomes of recently developed pH-responsive liposomes are summarized in Table 5.

### 5.3. Emerging Trends and Critical Challenges

Current research increasingly aims to expand pH-responsiveness toward multimodal and precision-oriented nanocarriers. Beyond single-trigger activation, a growing body of work demonstrates that coupling pH sensitivity with additional endogenous or exogenous stimuli enables synergistic, multistage control over drug release. Light/pH dual-responsive liposomes represent a prominent example, in which acidic conditions characteristic of the tumour microenvironment or endolysosomal compartments sensitize the carrier, while photoactivation provides remote and temporally controlled drug release. For instance, supramolecular systems integrating photo-responsive azobenzene/cyclodextrin complexes within pH-sensitive vesicular matrices have been shown to enable on-demand release under combined light and acidic pH stimuli, highlighting their potential for spatially and temporally controlled anticancer therapy [128]. Similarly, catanionic vesicles based on photoactive chalcone derivatives exhibit an AND logic-gated behavior, where significant drug release occurs only upon the simultaneous application of light irradiation and acidic pH, thereby enhancing spatial selectivity and minimizing premature drug leakage [129]. Comparable synergistic effects have also been reported in pH-responsive liposomes integrated with photothermal or temperature-sensitive components, where local hyperthermia induced by near-infrared irradiation accelerates membrane destabilization and drug diffusion in acidic environments, leading to improved cellular uptake and therapeutic efficacy [64].

These advances highlight the potential of coupling proton-triggered activation with additional environmental stimuli to achieve greater selectivity and treatment efficacy. Despite these promising developments, several challenges remain. Quantitative characterization of protonation-induced structural transitions is still limited, as many experimental studies lack real-time, molecular-level insights into bilayer reorganization. Moreover, the considerable variability in lipid composition, buffer systems, and analytical protocols across studies hampers direct comparison of reported pK_a_ values and release kinetics. Another persistent issue lies in balancing stability and responsiveness: liposomes that are too labile tend to release their contents prematurely, whereas overly stable formulations may resist activation under the mildly acidic conditions typical of tumour or endosomal environments. Recent constant-pH MD studies have begun to address the coupling of protonation states and membrane structure, although the methodology is still evolving [130]. Meanwhile, FTIR spectroscopy has been recently used to monitor pH-dependent protonation events at the lipid–water interface [131].

In summary, pH-responsive liposomes illustrate how molecular protonation equilibria can govern mesoscale organization and function. By coupling weakly acidic or basic lipids with polymeric or targeting modules, it is now possible to design vesicles that remain stable in circulation yet destabilize selectively in acidic microenvironments. Despite rapid progress, predictive modelling and systematic comparison across systems remain limited. Future work should focus on establishing standardized experimental parameters and reporting practices, including lipid ratios, pK_a_ ranges, and leakage kinetics, to improve reproducibility and enable predictive modeling of pH-responsive behaviour across different liposomal systems. Integrating quantitative spectroscopy, calorimetry, and simulation will be essential for developing next-generation nanocarriers that translate environmental pH gradients into precise therapeutic control.

## 6. General Conclusions and Outlook

Stimuli-responsive liposomal membranes represent a dynamic interface between molecular design and functional performance. Across thermal, photo-, and pH-triggered systems, a unifying theme emerges: controlled perturbation of bilayer organization through localized heating, light-induced isomerization, or protonation can reversibly modulate membrane permeability, curvature, and release kinetics.

Thermosensitive and photothermal formulations have achieved high precision in spatiotemporal control, particularly when plasmonic nanoparticles act as nanoscale heat transducers. Photochromic systems based on azobenzenes and spiropyrans demonstrate how purely molecular photoactuators can convert photon energy into mechanical responses, offering reversible, wavelength-selective regulation without inorganic additives. pH-responsive architectures complement these strategies by harnessing intrinsic biological gradients, providing autonomous activation and selective intracellular delivery.

While this review highlights common design principles underlying light-, temperature-, and pH-responsive liposomal membranes, a fully systematic quantitative comparison across different stimulus classes remains challenging. Variations in lipid composition, stimulus intensity and experimental conditions currently limit direct cross-comparisons. Bridging this gap requires the integration of quantitative structural probes (e.g., time-resolved SAXS, fluorescence lifetime imaging) with multiscale molecular simulations capable of correlating local events with collective behaviour. Standardized benchmarks, such as unified pK_a_ reporting and leakage assays, are also needed to ensure reproducibility across laboratories and facilitate quantitative benchmarking of responsiveness. Addressing these aspects will be critical for translating stimulus-responsive liposomal systems from proof-of-concept designs toward predictive, application-oriented nanomedicine.

Looking ahead, next-generation liposomes are expected to evolve into multifunctional, hierarchical systems in which distinct stimuli act sequentially or cooperatively. Combining light- or heat-responsive modules with pH-sensitive domains could enable multi-triggered release profiles, selective activation in diseased tissues, and precise subcellular targeting. The convergence of lipid chemistry, supramolecular design, and real-time imaging is likely to yield predictive control over membrane dynamics and unlock broader biomedical applications, from precision oncology to synthetic biology.

In essence, stimuli-responsive liposomes exemplify how subtle molecular reorganization can translate into programmable mesoscale function. Continued collaboration between experimental and computational disciplines will transform these systems from phenomenological tools into quantitatively engineered nanodevices, capable of operating as intelligent, adaptive components in future therapeutic and diagnostic platforms.

## Figures and Tables

**Table 1:** Summary of representative studies on gold nanoparticle–liposome hybrids for photothermal and thermo-responsive applications.

Reference/ System	Lipid Composition	AuNPs Configuration	Stimulus	Mechanism of Release/Effect	Key Findings	Limitations/ Remarks
[71] Liposome-templated AuNPs	DSPC	AuNPs nucleated on liposome surface (in situ core–shell)	NIR	Local photothermal heating/ controlled phase transition	Precise temp. control, effective tumour inhibition	Synthesis complexity; possible Au leaching; long-term stability not evaluated
[74] Liposome-tethered AuNPs	DPPC	AuNPs tethered via thiol-PEG to membrane	Pulsed NIR	Cavitation nanobubbles/transient pores-burst release	Rapid, efficient, repeatable release	Mechanical damage risk; limited quantitative ΔT data
[72] AuNP-decorated thermosensitive liposomes	DPPC/DDAB ±cholesterol	AuNPs anchored to bilayer surface	Heating	Phase transition-assisted release of DOX	Cholesterol content tunes permeability and stability; efficient drug delivery	High cholesterol may reduce heating efficiency; narrow T window
[73] PNIPAM—AuNP hybrid liposomes	DPPC	Thiolated-carboxymethyl cellulose-capped AuNPs inside bilayer	NIR and heat	PNIPAM collapse and photothermal heating	Dual-responsive release; reversible; good biocompatibility	Complex multi-component synthesis; limited cycle tests
[75] Effect of AuNP geometry on membranes	DPPC	AuNPs of different shapes	Thermal analysis	Structural perturbation	Rods and cubes induce stronger perturbation and higher uptake	No drug release data; model system only
[45] Hydrophobic AuNPs in SOPC bilayers	SOPC	Hydrophobic AuNPs inserted in bilayer core	Temperature ramp	Local membrane deformation	Demonstrated insertion stability and local disorder	No photothermal activation; relevance mainly mechanistic
[79] Effect of surface charge and rigidity	DOPC, DPPC, DOPS, DOTAP	AuNPs of varying surface charge interacting with liposomes	N/A (Passive interaction)	Structural and electrostatic effects	AuNPs interact more strongly with positively charged liposomes	Model study; no stimuli applied

**Table 2:** Representative azobenzene-based systems reviewed here, summarizing the formulation, optical trigger, observed functional outcome and the principal mechanistic insight.

Reference	System	Light Trigger (λ)	Release/Functional Effect	Key Mechanistic Insight
[88]	Cationic azo-lipid/DSPC liposomes (and UCNP co-loading in vivo studies)	UV/Vis activation; NIR via UCNP upconversion for in vivo	Enhanced doxorubicin release, endosomal destabilization, improved cytosolic delivery and tumour growth inhibition under NIR + UCNP	Azobenzene-driven molecular reorientation produces “nanomechanical” stresses on endosomal membranes; UCNPs extend activation into NIR for in vivo use.
[89]	POPC liposomes doped with aryl-azo derivatives of thymol	365/430 nm	Reversible modulation of membrane permeability; correlated Cl^−^ transport and dye release kinetics	Tunable permeability via small-molecule guest insertion
[91]	LipCal@NC—azobenzene nanoclusters tethered to liposomes	UV light	increased cumulative cargo release (~33 → ~47% after 360 min UV)	Cooperative action of clustered azobenzene units amplifies lipid disorder on isomerization
[92]	Mechanistic study: azobenzene embedded in lipid membranes	various (mechanistic study of 365/450 nm regimes)	N/A (mechanistic)	Demonstrates competing photoisomerization pathways in membranes; local packing and polarity shift the preferred pathway and affect quantum yields and fatigue.
[94]	Photosensitive liposomal formulations with systematically varied azobenzene substituents	UV/visible	Tunable release (reported examples: ~12–18% over 12 h, depending on substituent)	Electronic nature and charge of substituents strongly modulate photoisomerization efficiency in bilayers; positively charged/electrophilic groups can suppress switching yield.
[95]	Light-responsive nanoliposomes (azobenzene moiety) designed for NIR-operability (upconversion/sensitization strategies)	NIR (via upconversion/sensitized isomerization)	Controlled drug release under biologically compatible wavelengths	Use of upconversion or sensitization overcomes UV limitation; design balances photochemical efficiency and membrane confinement effects
[96]	Supramolecular photo-gated K^+^ channel (P3) incorporating azobenzene and β-cyclodextrin host–guest motifs	365/450 nm	Light-gated K^+^ transport in liposomes and cells; rapid cytosolic K^+^ depletion, ER stress, apoptotic cascades	Azobenzene cis-trans switching modulates intra/intermolecular H-bonding and host–guest complexation, producing multi-state (ON/Partially OFF/OFF) transport behaviour.
[97]	Azo-functionalized bambusuril chloride transporters (azo-BUs)	365/450 nm	Reversible light-tunable Cl^−^ transport; channel-like electrogenic behaviour; light-dependent cytotoxicity in cancer cells	E–Z photoisomerization gates channel-like conduction (electrogenic), disrupting ionic homeostasis and lysosomal acidification; single-molecule/channel-like behaviour observed.
[98]	Azobenzene-fused bis(1,3-diol) self-assembling chloride channels	365/450 nm	Photoresponsive Cl^−^/NO_3_^−^ antiport; conductance measurements support channel formation	Self-assembly into stable channel structures modulated by azobenzene isomerization; Hill coefficients ≈ 1 indicate discrete channel formation and light-regulated gating.

**Table 3:** Representative examples of spiropyran-based photoresponsive liposomal and hybrid membrane systems.

Reference	System	Light Trigger (λ)	Mechanism/Effect	Main Findings
[99]	POPC liposomes doped with SP	365/530 nm	Light-controlled proton translocation; intraliposomal acidification	SP → MC conversion drives migration to the interface; minimal but directional ion-gating cycle
[100]	SP-Cou-Cbl conjugate	Acidic pH + Visible light	Tumour-localization via fluorescence activation; controlled drug release	pH unlocks SP → MC and activates fluorescence; light triggers photorelease (“dual-key” mechanism)
[101]	Ternary supramolecular complex (SP derivative + Zn^2+^ + acetylsalicylic acid)	Visible light	Light-controlled release	Photo-responsive system enables light-controlled delivery of ASA and Zn^2+^ with reversible switching
[102]	UCNPs@SP-polymer nanocomposites	NIR + acidic pH	NIR-triggered release of DOX; enhanced cancer-cell killing	UCNPs convert NIR to UV to induce SP → MC; morphology disruption + pH-induced MC formation provides dual-stimulus release
[103]	SP-modified membrane-active peptide (BP100 analogs)	UV/Visible	Light-regulated membrane perturbation; control of cytotoxicity	SP → MC switching modulates peptide helicity, amphiphilicity, and membrane affinity; MC reduces activity
[104]	SP-containing cationic block copolymer	UV (365 nm)/Green (520 nm)	pH + light-triggered DOX release; Cu^2+^ sensing; high gene transfection	SP/MC switching tunes carrier polarity; MC enables Cu^2+^ detection; supramolecular assembly supports dual therapy + diagnostics
[105]	SP-crosslinked shell-cross-linked micelles (SCMs)	UV	Light-gated substrate selectivity in catalytic reactions	SP/MC conversion alters hydrophobicity in the cross-linked shell; acts as photo-gated transport layer (principle transferrable to drug delivery)

**Table 4:** Comparative overview of light-responsive liposomal systems based on azobenzene and spiropyran photoswitches.

Feature	Azobenzene-Based Systems	Spiropyran-Based Systems
Primary photochemical change	trans-to-cis geometric isomerization	SP (nonpolar) → MC (polar/zwitterionic) via C-O bond cleavage
Dominant membrane effect	Packing disruption, curvature stress	Interfacial polarity and hydration modulation
Typical light activation	UV-Vis (red/NIR possible with substitutions)	Visible-NIR (depending on substitution)
Response time/ reversibility	Fast, highly reversible	Moderate, reversible but slower
Photochemical fatigue	Low (high stability)	Relatively high (fatigue over multiple cycles)
Functional outcome	Strong permeability modulation, shape change	Reversible charge/polarity modulation, gentle release
Design challenges	Deep-tissue activation, aggregation control	Switching efficiency, stability under confinement

**Table 5:** Representative pH-responsive liposomal systems reported in 2024–2025, summarizing chemical triggers, functional mechanisms, and biological performance.

Reference	System	pH Trigger	Mechanism of pH Response
[120]	pH-responsive theranostic liposomes co-assembled with ruthenium oxygen probe and indocyanine green	Tumour extracellular pH	pH-enhanced membrane fusion and cellular uptake; pH sensitivity promotes tumour accumulation, while the Ru complex enables oxygen-sensitive imaging
[124]	DOPE-CHEMS Cisplatin Liposomes	pH 6.5–5.5	Acid-induced protonation of lipids promotes membrane destabilization
[125]	DOPE-based pH-sensitive liposomes coated with fucoidan	pH 5.5	Fucoidan–P-selectin targeting + acid-triggered endosomal escape; enhanced macropinocytosis and caveolin-mediated uptake
[126]	Glycopeptide-modified liposomes	pH 5–6	Acid cleavage exposes antigenic peptides, promoting cross-presentation
[127]	Hybrid pH-sensitive liposomes/extracellular vesicles	pH 7.4–5.0	Acidic pH induces liposomal destabilization and drug release; EVs maintain pH sensitivity.
[128]	Photo-/pH-responsive supramolecular vesicles (Azo-cyclodextrin in pH-sensitive matrix)	pH 4, 5, 6, 7.4 and 9.18	pH-responsive vesicle matrix combined with light-driven Azo–CD inclusion/exclusion enables dual-triggered drug release
[129]	Light- and pH-responsive catanionic vesicles (chalcone-based)	Strongly acidic pH (≈3.0)	pH-induced chalcone/flavylium cation conversion perturbs membrane charge and packing; effective release occurs only under combined pH and light stimuli

## Data Availability

No new data were created or analyzed in this study.
